# 

**DOI:** 10.1192/bjb.2024.65

**Published:** 2025-02

**Authors:** Seamus O'Mahony

**Affiliations:** Glasgow End of Life Studies Group, University of Glasgow, UK. Email: enquiries@seamusomahony.com

**Figure:**
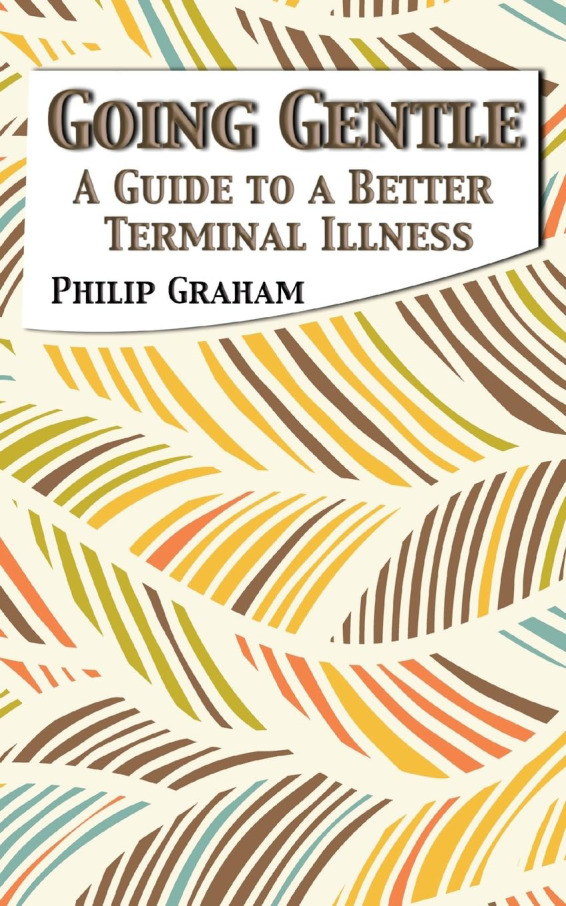

Philip Graham (born 1932) is Emeritus Professor of Child Psychiatry at the University College London (UCL) Great Ormond Street Institute for Child Health, London, UK, and was a consultant child and adolescent psychiatrist at Great Ormond St Hospital, London, UK, for over 25 years. He has written a short *ars moriendi*, *Going Gentle: A Guide to a Better Terminal Illness.* (The title is, of course, a nod to Dylan Thomas's ‘Do not go gentle into that good night’.) The book combines Graham's personal history and opinions with the experiences of the surviving families and spouses of 24 of his deceased friends. The editor of the Obituaries section of the *BJPsych Bulletin*, the author has reached an age when many, if not most, of his friends and contemporaries have died. These friends are a highly select group (they include Sir Jonathan Miller and Baroness Mary Warnock); half of them were honoured with an obituary in a national newspaper. But, as he wryly observes, ‘[w]hen the rich are dead, they are just as dead as the poor’ (p. 5).

Graham had his own brush with mortality at the age of 59, when he underwent surgery for intussusception in a small Swiss hospital. Because of poor post-operative fluid management, he developed acute pulmonary oedema, and had to be airlifted back to the shabby but competent Whittington Hospital in London, UK, where he made a swift and complete recovery. During this illness, which lasted about a month, Graham considered the possibility of death, and asked himself a series of questions on life, death and religious faith: ‘I came to few definite conclusions but felt calmer for having tried’ (p. 13).

The first section of the book grapples with these fundamental questions, and then takes a sharp turn in a more practical direction. Graham covers the pathophysiological details of terminal illness and (based on the feedback from the families of his dead friends) how end-of-life care might be optimised and the chances of a bad death minimised. There is much practical information here, particularly for readers who do not have a healthcare background. Some of these chapters will, inevitably, be of less interest to medical readers, but, as he astutely observes, ‘[m]ost even highly educated people have very little idea of the way their bodies function’ (p. 3).

Graham is a former chair of Dignity in Dying and has predictably trenchant views about assisted suicide. He attributes hospice physicians’ opposition to assisted suicide partly to Dame Cicely Saunders’ strong Christian faith: ‘Although palliative care physicians are now much less likely to share her Christian beliefs, there remains a religious element to the movement.’ I don't think this is the case, but that is to cavil. This book is a wise, humane and practical guide to that journey we all must face.

